# PV Defects Identification through a Synergistic Set of Non-Destructive Testing (NDT) Techniques

**DOI:** 10.3390/s23063016

**Published:** 2023-03-10

**Authors:** Socrates Kaplanis, Eleni Kaplani, Paul Nicolae Borza

**Affiliations:** 1 Renewable Energy Systems Lab, University of Peloponnese, 26334 Patra, Greece; 2 Lab of Soft Energy Applications and Environmental Protection, University of West Attica, 12201 Athens, Greece; 3 School of Engineering, Faculty of Science, University of East Anglia, Norwich Research Park, Norwich NR4 7TJ, UK; e.kaplani@uea.ac.uk; 4 Department of Electronics & Computers, Transilvania University of Brasov, 500036 Brasov, Romania; borzapn@unitbv.ro

**Keywords:** PV defects and diagnostics, NDT techniques, electroluminescence, UV fluorescence imaging, IR thermography

## Abstract

A synergistic set of NDT techniques, including I–V analysis, UVF imaging, IR thermography, and EL imaging, supports a diagnostics methodology developed in this work to qualitatively and quantitatively identify a wide range of PV defects. The methodology is based on (a) the deviation of the module electrical parameters at STC from their nominal values, for which a set of mathematical expressions was developed that provide an insight into potential defects and their quantitative impact on the module electrical parameters, and (b) the variation analysis of EL images captured at a sequence of bias voltages for a qualitative investigation on the spatial distribution and strength of the defects. The synergy of these two pillars, supported by UVF imaging, IR thermography, and I–V analysis cross-correlating their findings, makes the diagnostics methodology effective and reliable. It was applied on c-Si and pc-Si modules operating from 0–24 years, exhibiting a diversity of defects of varying severity, either pre-existing or formed by natural ageing or externally induced degradation. Defects such as EVA degradation, browning, corrosion in the busbar/interconnect ribbons, EVA/cell-interface delamination, pn-junction damage, e^−^+hole recombination regions, breaks, microcracks, finger interruptions, and passivation issues are detected. Degradation factors triggering a cascade of internal degradation processes through cause and effect are analysed and additional models are proposed for the temperature pattern under current mismatch and corrosion along the busbar, further empowering the cross-correlation of NDT results. Power degradation was determined from 1.2% in 2 years of operation to more than 50% in modules with film deposition.

## 1. Introduction 

The photovoltaic (PV)-ageing and performance-degradation modes have been extensively studied as far as they concern weathering due to outdoor conditions [1,2,3], early ageing [4] and long-term operational degradation [5,6]. Indoor ageing and thermal-cycling cumulative exposure were previously studied to predict degradation in the series resistance R_s_ [7] and optical degradation [8]. Reports on sensors, non-destructive testing (NDT), and methodologies focus on the diagnosis of ageing factors and their effects [9,10,11,12,13], whereas analysis of faults linked to degradation factors and defects and their implications are provided in [14,15,16,17,18]. External factors, such as solar radiation, shading, wind, and other environmental parameters, as well as internal factors, along with the conditions that trigger degradation processes, have been investigated in depth. Strong PV-cell temperature T_c_, fluctuations, or cycles may cause thermal shocks that trigger ageing processes and/or structural deformation [1,2,6,7,9,13]. Ambient temperature T_a_ cycles in cold sites or in deserts cause module thermal fatigue and damage the frame, similarly to the strong wind effect, resulting in cracks, frame deformation, and enhancement of humidity ingress [19,20,21]. Various degradation factors related to ethylene–vinyl acetate (EVA) ageing, back-sheet delamination, and thermal cycling were studied in [22,23,24,25]. The impact of degradation factors on the PV modules’ electric parameters was analysed in [26,27,28], whereas a review on the PV-degradation rates is provided in [29]. PV-cell defects due to manufacturing failures or local or peripheral conductivity paths acting as shunts were also investigated, demonstrating that deleterious atoms left during doping act as recombination centres, i.e., shunt diodes, and affect the diode ideality factor m [9,30,31,32]. 

NDT tools such as I–V curve analysis, electroluminescence (EL), IR thermography, lock-in thermography, photoluminescence (PL), UV fluorescence (UVF), digital-image processing, and Raman spectroscopy were used for the identification of internal and external degradation factors and defects [30,31,32,33,34,35,36,37]. In the EL phenomenon, when current is fed into a module under positive-bias voltage V, photons of λ = 1150 nm are emitted in Si-based cells. This is because, along their paths in the semiconductor, electrons undergo a series of sub-processes, such as energy-level transitions and/or e^−^+hole recombination. Thus, the electron paths may be practically traced. The emitted light intensity and its spatial distribution provide information about the cells’ health status, the quality of the manufacturing process, shunt defects, series resistance, cracks, grid-line interruptions, or inactive parts in cells. UVF images are characterised by the intensity and spatial distribution of the fluorescent light emitted by various chromophores. These are created from the dissociation of EVA molecules under the UV action when a considerable amount of solar-radiation dose (SRD) (MWh/m^2^) is absorbed by the PV cells during the years of operation [38,39,40]. The excitation of the fluorescing molecules is achieved by a UV lamp or laser. The fluorescence is radiated at longer wavelengths and is detected by a camera equipped with a band-pass filter above 400 nm where the peaks of the chromophores are. UVF imaging may be used to easily detect EVA degradation. IR thermography discloses T_c_ patterns and hotspots caused by internal or external factors. The IR-tool effectiveness increases when the module is in operation, and so both the T_c_ and the radiated heat are high. IR thermography is a useful, simple, and fast technique to detect hotspots due to corrosion and other defects [41,42,43].

This paper aims to build and assess a methodology of PV-defect diagnosis based on a synergistic set of NDT tools cross-correlating their findings, as the existing methods independently cannot effectively cover the full range of defects. The methodology proposed is based on (1) the analysis of deviations of the module electrical parameters at standard test conditions (STC) from their nominal values and (2) the variation analysis of EL images captured under various bias voltages. The methodology is further supported by optical inspection, IR thermography, UVF imaging, and I–V analysis. Furthermore, it aims to provide in-depth understanding of the development of avalanches of cause-and-effect processes that trigger the formation of defects. The effect caused by internal and external degradation factors is quantified, and that provides a thorough picture of the PV defects, their impact, and their evolution, where T_c_ plays the role of the cause and/or of the effect. The methodology proposed has been applied to c-Si and pc-Si modules, from those that are brand new to those that have been operating for 2, 4, 5, 18, and 24 years subjected to induced and natural degradation during their lifetime. The synergy of NDT techniques is shown in this paper to enhance the effectiveness of the defect diagnosis. 

## 2. Identification of Defects, Cause and Effect, and the Need for Synergistic NDT Tools

This section presents an in-depth investigation into PV defects and identification techniques using NDT tools, which are applied in a large number of c-Si and pc-Si modules, providing important information concerning the defects/degradation factors, their cause-and-effect chain, and the synergy required in the NDT tools.

**Figure 1:**
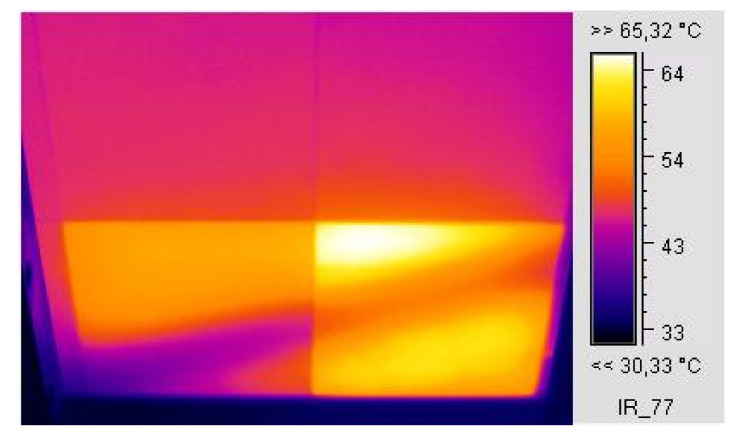
IR thermography of a pc-Si module captured at the backside of the module, showing a transient shadow falling diagonally on the two bottom cells, creating temporary T_c_ patterns in the affected cells.

**Figure 2:**
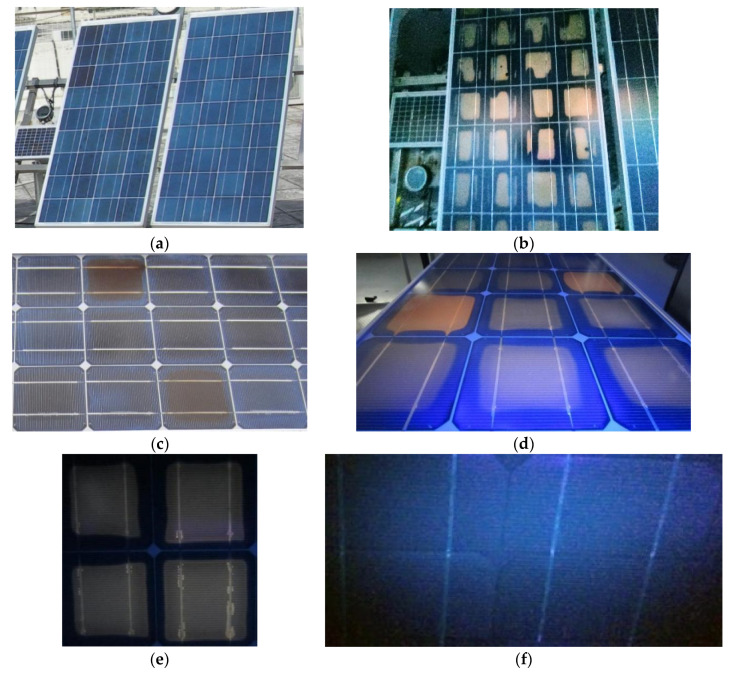
(**a**) pc-Si ES120 modules shows early signs of EVA browning, (**b**) UVF image of the modules in (**a**) reveals EVA degradation, (**c**) c-Si M55 module showing cells with various levels of browning, (**d**) UVF image of the module in (**c**) revealing different degrees of EVA degradation, (**e**) UVF image of a c-Si M55 module shows EVA browning and delamination of EVA/cell interface, (**f**) UVF image of a c-Si AP50 module exposed to low levels of SRD without signs of EVA degradation.

External factors such as patterns of shadows and inorganic- or organic-film depositions on a module cause inhomogeneities in the distribution and transmission of the incident solar irradiance I_T_ into the cell/module. These may act as degradation factors with direct and/or indirect effects on the I–V, the cell electric parameters, the PV performance, and ageing [33,44,45,46,47]. The result may either be a uniform current I decrease, when the strength of their effect is the same in all cells, or current mismatch, that is, a decrease in I by δI when the shading or the layer deposition is not uniform or the light transmissivity differs from cell to cell. δI is converted into heat due to voltage polarisation across the cells connected in series, which appears macroscopically as a T_c_ pattern higher than in the unaffected cells. In the experiments carried out, T_c_ patterns of various levels appeared when pc-Si cells were partially shaded, as shown in the IR image (Figure 1), where current mismatch was the generating cause. This process was temporary and did not result in permanent degradation. Generally, both mismatch and the associated T_c_ pattern cause I–V distortion, and provided the cause lasts for a long time, it may result in EVA degradation and browning to various degrees, as shown in the digital and UVF images (Figure 2a–d). Long-standing high T_c_ patterns caused EVA discolouration or browning in alignment with the high SRD or the UV effect on the cells in pc-Si modules operating for 8 years on a double-axis sun-tracking system (Figure 2a,b). Strong mismatch and associated high permanent T_c_ patterns were primarily developed after lumps of mortar were shed on the PV array, leaving several cells fully or partially covered for a prolonged period of several months in the Renewable Energy Systems Lab, Greece. After cleaning the cells, EVA degradation, browning, current mismatch, and high T_c_ patterns remained. The cause of the high T_c_ was the non-uniform I_T_ transmission in the module due to various degrees of EVA browning in cells (Figure 2c,d). The latter caused permanent current mismatch. This is an aftermath effect that causes second-stage/-order defects. Generally, irreversible physicochemical changes in the EVA and cell may develop and expand, e.g., EVA degradation, browning, corrosion, or EVA/cell-interface delamination, as shown in Figure 2c–e.

In general, PV-cell shading for a prolonged amount of time may become the forerunner of time-evolving EVA degradation or ageing, and T_c_ patterns may appear due to heat dissipation in the affected cells (Figure 1). When the shading is transient the effect is reversible and the ageing follows a natural low progression.

**Figure 3:**
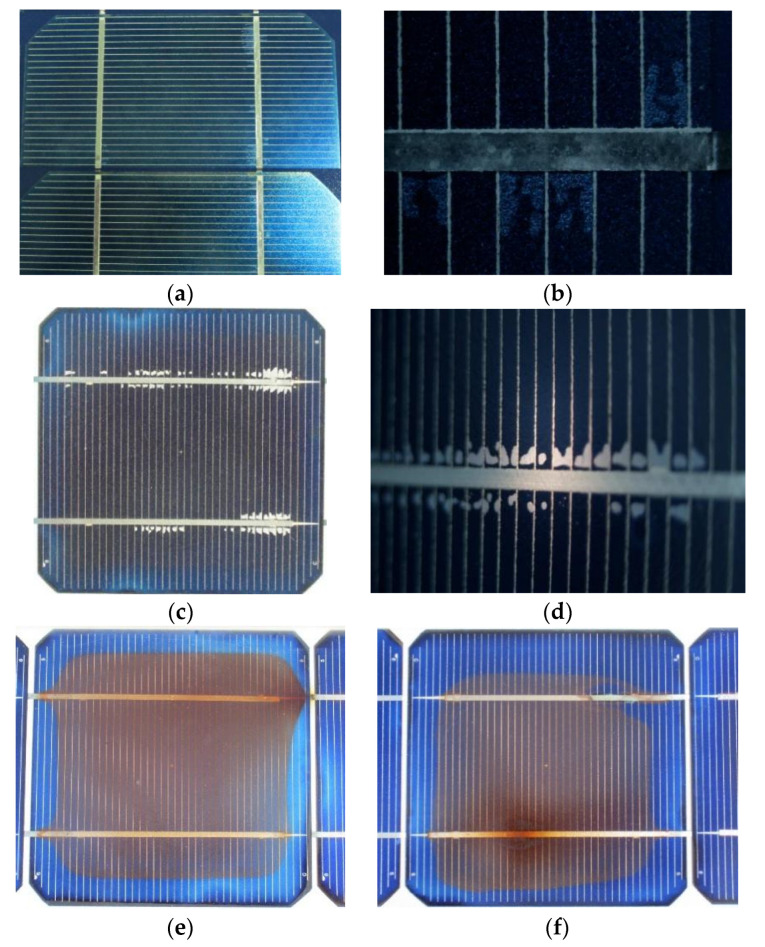
(**a**) Digital image and (**b**) microscopy (20×) of c-Si cells of an AP50 module exhibiting early signs of delamination in the EVA/cell interface, (**c**) c-Si cell of an M55 module with extended delamination in the EVA/cell interface along the busbar, (**d**) microscopy (10×) of a c-Si cell in the same module showing similar delamination along the busbar, (**e**,**f**) digital images of M55 cells with severe corrosion on the busbar and interconnect ribbon due to high T_c_ developed there and EVA-molecule dissociation.

As disclosed in this project, cells with extensive EVA browning cause permanent current mismatch, leading to high T_c_, or hot cells, which degrade or age the physicochemical status of the cells at a fast rate. These may trigger a cascade of ageing factors, bringing the cells into an irreversible state and causing, in turn, further permanent physicochemical changes and high permanent T_c_ patterns, as also reported in other studies [7,11,48]. EVA degradation is usually linked to exposure to high SRD, whereas negligible EVA degradation is expected in cells exposed to low SRD. The UVF image (Figure 2f) of a c-Si module exposed to low SRD (0.2 MWh/m^2^) displayed no signs of EVA degradation; however, early signs of delamination in the EVA/cell interface along the busbar were present (Figure 3a,b). This appeared to be at the EVA/ARC interface. Severe EVA/cell-interface delamination (Figure 3c,d) and corrosion on the busbar and interconnect ribbon along with EVA browning (Figure 3e,f) were present in a c-Si module exposed to SRD = 48 MWh/m^2^. Adhesive degradation at the EVA/silver interface has been shown to be responsible for the delamination around the cell’s metallisation [49,50]. EVA degradation linked to the formation of acetic acid and weakening of the adhesion through the high T_c_ developed may also result in corrosion.

### 2.1. IR Thermography for the Identification of the Nature of Degradation

#### 2.1.1. Current Mismatch and Temperature Effect

The current decrease δI in a shaded cell multiplied with the polarisation voltage developed across the cell V_oc,c_(n − 1) and the voltage across the diode V_d_ provides the power dissipating into heat which contributes to a further increase in temperature of the shaded cell above the healthy cells’ temperature due to operation under certain conditions. The heat developed in the shaded cell is then transferred to the environment, as described by Equation (1a). V_oc_,_c_ is the cell open-circuit voltage, n is the number of cells protected by the bypass diode, A_c_ is the area of the solar cell, and η is the module efficiency.

(1a) $$\delta I\left(V_{\mathrm{oc},c}\left(n-1\right)+V_{d}\right)+\left(1-\eta \right)A_{c}{Ι}_{Τ}={\left(h_{c}+h_{r}\right)}_{f}A_{c}\left(T_{f}-T_{a}\right)+{\left(h_{c}+h_{r}\right)}_{b}A_{c}\left(T_{b}-T_{a}\right)$$

(1b) $$\delta I\left(V_{\mathrm{oc},c}\left(n-1\right)+V_{d}\right)+\left(1-\eta \right)A_{c}{Ι}_{Τ}=({\left(h_{c}+h_{r}\right)}_{f}+{\left(h_{c}+h_{r}\right)}_{b})A_{c}\left(T_{\mathrm{pv}}-T_{a}\right)$$

(h_c_ + h_r_)_f_ stands for the heat convection and the radiated-heat coefficients for the front side, whereas (h_c_ + h_r_)_b_ is for the back side of the cell. T_f_ and T_b_ stand for the temperatures at the front glass and back side of the PV, respectively [51]. Equation (1a) can be simplified to Equation (1b), assuming T_f_ = T_b_ = T_pv_, even though up to a 3 °C difference may exist between T_f_ and T_b_.

**Figure 4:**
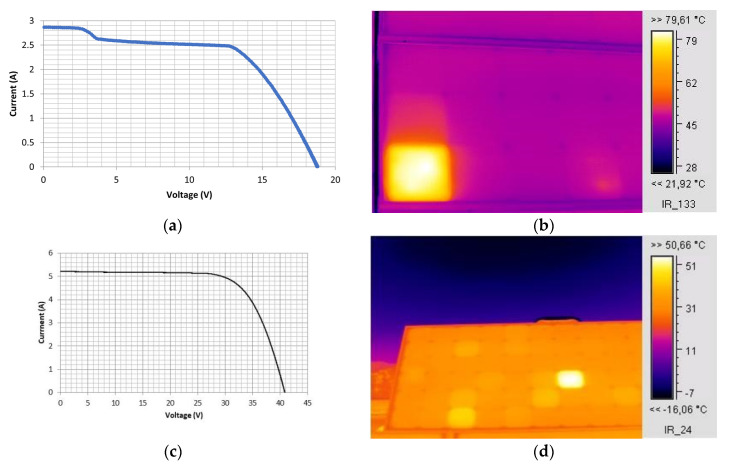
(**a**) The I–V characteristic and (**b**) IR thermography of an M55 module, operating for 24 years, with EVA browning in the bottom-left-corner cell. The affected cell exhibits increased temperature of around 34 °C higher than neighbouring cells in the module. (**c**) I–V curve of a brand-new c-Si BIO175 module at I_T_ = 899 W/m^2^, (**d**) IR image of the same module during operation.

The EVA browning in one of the cells of the c-Si M55 module created an effect of partial shading, leading to a current drop of 0.25 A in the operating conditions, as shown in the I–V characteristic (Figure 4a), which in turn led to the affected cell operating at a higher temperature by about 34 °C from the neighbouring unaffected cells of the module, as shown in the IR thermography (Figure 4b). The module I–V characteristic was measured using I–V curve analyser PV-KLA (Ingenieurbüro Mencke & Tegtmeyer GmbH, Hameln, Germany) and the IR thermography was captured using an IR camera IC085LV (TROTEC GmbH, Heinsberg, Germany). Equation (1b), for n = 18 and V_d_ = 0.6 V and considering (h_c_ + h_r_)_f_ +(h_c_ + h_r_)_b_ about 20 W/m^2^K at the conditions of the experiment v_w_ = 2.1 m/s, I_T_ = 760 W/m^2^, and T_a_ = 30.9 °C, predicted the temperature of the affected cell T_pv_ = 74 °C, which is in agreement with the IR thermography (Figure 4b) showing the affected cell reaching temperatures between 74–79 °C. This explains that the PV temperature pattern developed was due to the current mismatch caused by the cell exhibiting EVA browning.

Milder thermal effects linked to current mismatch were also identified in new modules. The IR image of a brand-new c-Si BIO 175 W_p_ under operation revealed a hot cell with T_c_ over 15 °C higher than the temperature of the neighbouring cells in the module (Figure 4d). This is attributed to localised imperfections at the production stage and may signal the onset of degradation. Figure 4c shows the module’s I–V characteristic, where δI dropped by 0.03 A in the first 6 V due to associated current mismatch. Such defects are pre-existing and trigger mild degradation.

The above confirms that the hot cells detected via IR thermography were linked to current-mismatch effects. The origin of high temperature differences observed between individual cells and their neighbouring cells, are generally due to current mismatch caused by localised EVA browning, whereas smaller temperature differences may indicate localised shading if the phenomenon is transient; otherwise, indicate the existence of cell microdefects caused during the manufacturing stage, which may become the onset of permanent degradation.

#### 2.1.2. Hotspots Linked to Corrosion 

**Figure 5:**
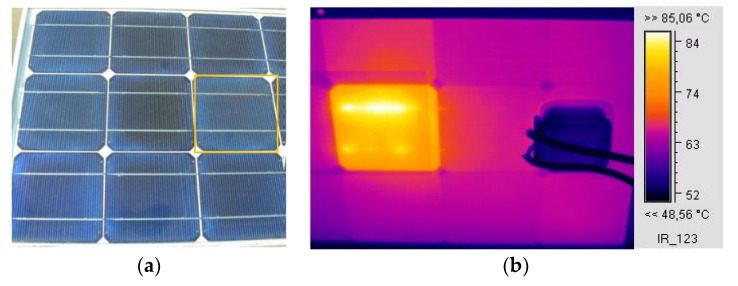
(**a**) Digital image from part of a 53 W_p_ c-Si module with visible EVA and ARC degradation. (**b**) IR image captured from the back of the module showing hotspots on the busbars of the cell indicated in (**a**).

A case where EVA degradation led to corrosion at the busbars is shown in Figure 5a,b. The affected cell appeared upon close visual inspection to have mild EVA and ARC degradation similar to neighbouring cells. The IR image (Figure 5b) revealed hotspots at the busbars, which may have been due to corrosion, with a temperature 20 °C higher than that of neighbouring cells. 

Corrosion may cause a significant increase in the *R_s_*. The latter, due to the Joule effect, increases the T_c_. A certain amount of power I^2^δR_s_(W) is converted into heat and transferred to the environment at a rate of 2(h_c_ + h_r_)A_cor_δT_c_. Therefore,

(2) $$I^{2}{\delta R}_{s}=2\left(h_{c}+h_{r}\right)A_{\mathrm{cor}}{\delta T}_{c}$$

where (h_c_ + h_r_) was estimated equal to 12.5 W/m^2^K, the area affected by corrosion A_cor_ was estimated from the area of the hotspots along the busbars to equal 2.45 cm^2^, the current at operating conditions (800 W/m^2^) was estimated to be I = 2.4 A (I = 3 A at 10^3^ W/m^2^), and the measured ${\delta T}_{c}$ was 20 °C higher than the temperature of the neighbouring cells. These lead to a ${\delta R}_{s}=0$.021 Ω or 21 mΩ higher than the $R_{s}$ of neighbouring cells of the module. Hotspots identified through IR thermography indicated resistive busbars or interconnects attributed to corrosion often co-existing with EVA degradation or humidity ingress. 

### 2.2. UVF Imaging for the Detection of EVA Degradation 

**Figure 6:**
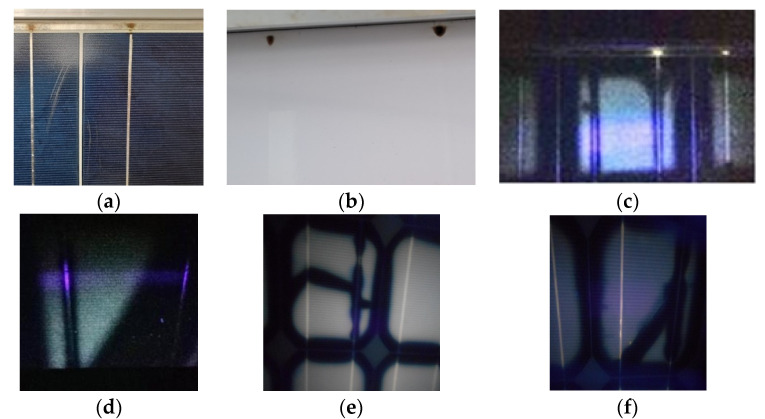
(**a**) Digital image of the front and (**b**) back side of the large pc-Si ODT no1 showing 2 corrosion signs. (**c**) The UVF image clearly shows both corrosion signs and suppression of the UV fluorescence in the cell edges and the busbar. (**d**) Diagonal UV fluorescence pattern, (**e**,**f**) UVF images of a SW80 module showing invisible cracks. UVF images captured during PV illumination with UV light at 375 nm.

Corrosion in the busbar and the interconnect ribbon developed due to the acetic acid formed by EVA photodegradation at high T_c_ (Figure 3e,f). Under natural ageing and an SRD of around 8 MWh/m^2^, corrosion formed on the interconnect ribbon in one of the pc-Si modules. Figure 6a,b shows two signs corrosion, associated with humidity ingress, near the T connection between the busbar and the interconnecting ribbon at the front and back side of the ODT-660P module. The UVF image of the ODT module (Figure 6c) shows signs of corrosion and EVA degradation. The EVA degradation pattern was symmetrically shaped and contoured by a non-UV-fluorescing area because O_2_ diffused through the back sheet and suppressed the fluorescence and photo-bleaching along the cells edges, which also appeared along the busbars. Figure 6d shows a complex pattern of EVA degradation revealed through UVF imaging. The bleached area along the busbar was attributed to the weakening of the cell metallisation, which eased O_2_ diffusion from the back side. Bleaching appeared in the diagonal part of the cell, which showed a non-UV-fluorescent pattern possibly due to bending caused by high wind loads impacting the large pc-Si ODT surface. Hence, back-sheet delamination started and O_2_ diffused into the affected cells. Such patterns did not appear in the UVF images of the smaller M55 modules, which supports the above hypothesis. For the smaller c-Si M55 modules, signs of corrosion were evident at SRD > 24 MWh/m^2^.

The UVF image reveals the extent of EVA degradation. The intensity of the fluorescence from the EVA degradation pattern was proportional to the number of chromophores due to the dissociation of EVA molecules by the UV of the SRD, as also supported by Figure 2b,d–f. Signs of EVA browning appeared in pc-Si ES modules 124 W_p_ under natural ageing conditions at SRD > 10 MWh/m^2^, as shown in Figure 2b. The EVA degradation patterns in small c-Si modules follow the cell geometry. In large modules, the patterns may have non-symmetric shapes, as in Figure 6d, where the bleached area was diagonal due to the stress loads that bent the module. Figure 6e,f show UVF images of c-Si SW80 cells that suffered from non-visible cracks.

Although UVF imaging may detect EVA browning even at the early stages of formation, often the latter appears to develop alongside other defects, which gives rise to the need for combined diagnostics. I–V analysis and IR thermography are required to provide more details on the effect of EVA degradation in its different modes, combined with EL imaging on the extent of non-visible cracks, e^−^+hole recombination centres, R_s_, and R_sh_.

### 2.3. Delamination of EVA/Cell Interface at the Metallisation 

**Figure 7:**
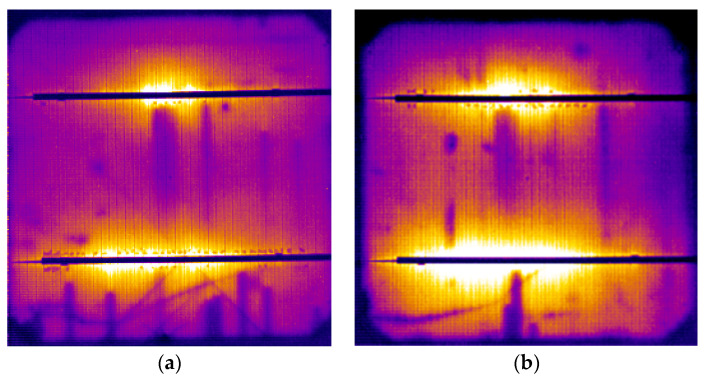
(**a**,**b**) EL images (pseudocolour) of two cells of an M55 module showing darker spots at the delaminated areas around the busbar. Module forward biased at 19.5 V and 19.8 V, respectively.

Delamination of the EVA/cell interface around the busbar was observed in several cells of the M55 modules operating for 18 and 24 years, such as the ones shown in Figure 3c,d. EL tests using the short-wave infrared (SWIR) camera Goldeye-P008 (Allied Vision Technologies, Germany) were performed on a M55 module with the module forward biased at 19.5 V–19.8 V (V_oc_ = 21.7 V). The resulting EL images (Figure 7a,b) revealed darker contrast in the delaminated spots around the busbar, whereas the surrounding area was EL bright due to e^−^+hole recombination. Delamination of the EVA/cell interface caused a small decrease in the current proportional to the delaminated area, a small increase in the R_s_, and a decrease in the R_sh_. The effect can be quantified through I–V analysis at the cell level. As δR_s_ is usually very small, the T_c_ profile along the delaminated area was very small, too, and the IR thermography did not detect any essential T_c_ pattern. The delamination spots formed during the years of operation expanded in the x–y dimension, whereas new spots appeared whose rate depended on the SRD and the level of the T_c_ developed. High-resolution EL imaging enabled the investigation of the impact of delamination at the EVA/cell interface. Other defects, such as broken fingers and shunts, were also visible. The combination of a wide range of degradation effects that can be detected through EL imaging gives rise to the need for a combination with other techniques for the quantification of the degradation caused by the individual defects. 

### 2.4. Corrosion in Cables and Identification by I–V Analysis

**Figure 8:**
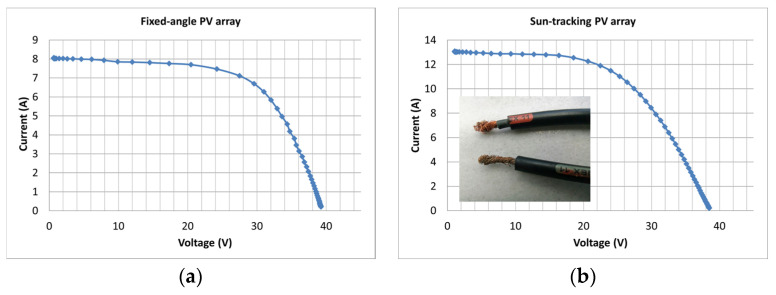
The I–V characteristics of two identical PV generators (**a**) at a fixed angle and (**b**) sun-tracking. The photo in (**b**) shows corrosion in one lead of the cable.

I–V analysis can assist with the quantification of degradation from a wide range of factors and defects, even in cases where corrosion has occurred in the cables, which can be missed from module-oriented IR thermography. Corrosion in a cable connected to the junction box was identified through comparative I–V analysis in two identical PV generators, one fixed and one sun-tracking. The I–V characteristics are shown in Figure 8a,b. The V_m_ in the sun-tracker (Figure 8b) compared to the fixed PV (Figure 8a) was shifted by 3.5 V, whereas the shift due to their T_c_ difference was estimated at 1.8 V. Therefore, the V_m_ and P_m_ decreases in the sun-tracking system could not be interpreted by the T_c_ effect alone. The additional δR_s_ was attributed to a hidden corrosion in the cable, since cracks, delamination, or visible corrosion contributing to δR_s_ were not detected by IR, EL, or UVF. 

Although certain defects can be identified by more than one technique, often a single technique is not sufficient to diagnose the nature of a degradation effect. The cross-correlation of findings between the different NDT tools promises a more sensitive and accurate defect diagnosis.

## 3. The Defect Diagnostics Using Cross-Correlation of the NDT Findings: Results, Analysis, and Discussion 

The methodology developed consists of two main components: (1) analysis of the deviations of the module electrical parameters V_oc_, V_m,_ I_sc_, I_m_, R_s_, and R_sh_ at STC from their nominal values, and (2) the study of the variations of EL images captured at various bias voltages while measuring the current allowed into the module. A detailed study to identify cell defects and degradation factors and their effect on the power performance was carried out by cross-correlating findings from I–V analysis and EL, IR, and UVF imaging. 

The PV-defect-diagnostics methodology was applied to two groups of modules: (1) c-Si M55/SM55 and (2) pc-Si ODT-660P, with three modules in each group. The nominal values are given in Table 1. 

The aforementioned modules, with different years of operation for each one, were electrically characterised. The I–V characterisation was performed using GTM-AAA Flash Tester (Keyland, Jiangsu, China), including the I–V tracer, combined with a synchronized PV-temperature-monitoring unit. PV modules were exposed to simulated solar light at 1000 W/m^2^ at room temperature. Adjustments for any differences in measured PV temperature from T_c_ = 25 °C were carried out through conversion of the I–V characteristic to STC, according to the I–V translation equations in [52]. The electrical parameters of the modules V_oc_, V_m,_ I_sc_, I_m_, R_s_, and R_sh_ were then easily extracted from the converted I–V characteristic at STC. R_s_ was determined from one I–V characteristic based on [53]. The electrical parameters expressed at STC were then compared to the nominal values to determine the experimental deviations δV_oc_, δV_m,_ δI_sc_, δI_m_, δR_s_, and δR_sh_. In the first component of the methodology, the deviations were also predicted by the equations proposed in Section 3.1, and any differences observed between predicted and experimental deviations of the electrical parameters provided important insights into the electrical parameter(s) mainly responsible for the observed degradation and, indirectly, the nature of the defect.

In the second component, a series of EL images was captured in the dark with each module forward biased at a sequence of bias voltages while monitoring the current allowed into the module. The EL image capture was performed using the Module Fault Tester (Keyland, Jiangsu, China). The EL testing unit utilises a cooled near-infrared CCD camera. The EL images at different bias voltages revealed significant qualitative information on the nature of the defects, which were quantified by the I–V analysis and the analysis of deviations of the module electrical parameters (first component of the proposed methodology). The methodology is described analytically in Section 3.2, along with its application in the two groups of modules, with the main findings reported. 

Certain degradation effects revealed through either of the two components of the proposed methodology may need to be cross-checked with IR thermography and UVF imaging where necessary for the final sorting. This highlights the synergistic nature of the proposed NDT methodology. 

### 3.1. The Proposed Diagnostics Component Based on the Deviation Analysis of the Module Electrical Parameters: Results and Analysis

The methodology for defect identification is based on the following equations, which determine the deviations of the module electrical parameters at STC from their nominal values.

An expression of the V_oc_ deviation δV_oc_ due to a change in Ι_sc_. δΙ_sc_ is given by Equation (3), which is derived from Equation (4) for constant T_c_ and I_o_.

(3) $${\delta V}_{\mathrm{oc}}=n_{s}m\left(\frac{{\mathrm{kT}}_{c}}{q}\right)\left(\frac{1}{I_{\mathrm{sc}}}\right){\delta I}_{\mathrm{sc}}$$

(4) $$V_{\mathrm{oc}}=n_{s}m\left(\frac{{\mathrm{kT}}_{c}}{q}\right)\ln\left(\frac{I_{\mathrm{ph}}}{I_{o}}+1\right)≅n_{s}m\left(\frac{{\mathrm{kT}}_{c}}{q}\right)\ln\left(\frac{I_{\mathrm{ph}}}{I_{o}}\right)≅n_{s}m\left(\frac{{\mathrm{kT}}_{c}}{q}\right)\ln\left(\frac{I_{\mathrm{sc}}}{I_{o}}\right)$$

where n_s_ is the number of cells in the series, I_ph_ the photocurrent, I_o_ is the dark saturation current, k is the Boltzmann constant, and q is the electron charge.

In general, T_c_ and I_o_ are not constant, as assumed in Equation (3). Note that in cases of cells with EVA browning or busbar corrosion, a high δT_c_ pattern is developed in those cells identified through IR thermography. Introducing the effect of the deviations of T_c_ and I_o_ into Equation (4) yields Equation (5).

(5) $${\delta V}_{\mathrm{oc}}=n_{s}m\left(\frac{{\mathrm{kT}}_{c}}{q}\right)\left(\frac{{\delta I}_{\mathrm{sc}}}{I_{\mathrm{sc}}}-\frac{{\delta I}_{o}}{I_{o}}\right)+n_{i}m\left(\frac{{k\;\delta T}_{c,i}}{q}\right)\ln\left(\frac{I_{\mathrm{sc}}}{I_{o}}\right)+n_{j}m\left(\frac{{k\;\delta T}_{c,j}}{q}\right)\ln\left(\frac{I_{\mathrm{sc}}}{I_{o}}\right)+\ldots$$

where n_i_, n_j_, etc. denote the number n_i_ of cells with T_c_ higher than 25 °C by δT_c,i_ due to any defects. This is similar for the group of cells n_j_, etc.

**Figure 9:**
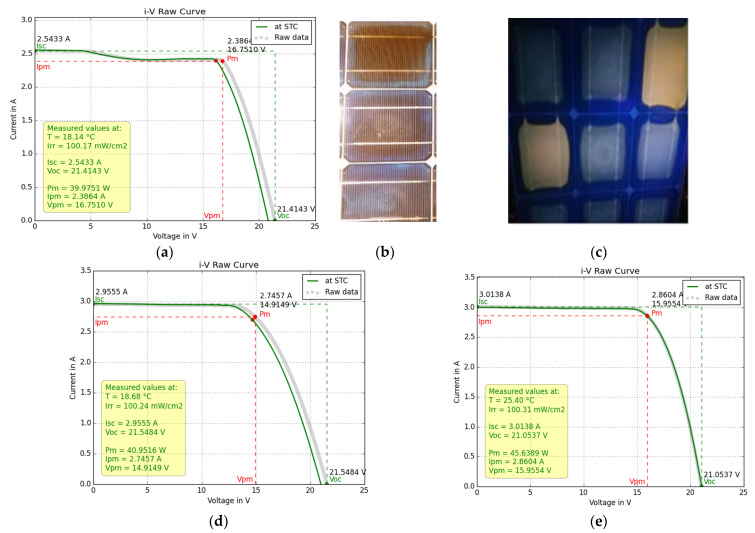
(**a**) I–V characteristic of module no1 captured with the flash tester and converted to STC, (**b**) digital image of the same module showing many cells with severe EVA browning, (**c**) UVF image of same module showing 2 cells with severe EVA browning and neighbouring cells with natural EVA degradation, (**d**) I–V characteristic of M55 module no2, and (**e**) I–V characteristic of SM55 module no3.

The I–V characteristic of M55 module no1 captured with the I–V flash tester and converted to STC is shown in Figure 9a. The decrease of δI_sc_ = 0.80 A from its nominal value (Table 1) was attributed to extended EVA browning (Figure 9b,c). A gradual decrease of 0.15 A in I yielded a total δI_sc_ = 0.95 A. Substituting the above values and (kT_c_/q) = 0.026 V, m = 1.5 (due to the recombination effect on the ideality factor, m), and n_s_ = 36 into Equation (3) yielded δV_oc_ = 0.525 V, which is less than the experimentally determined δV_oc_ = 0.88 V obtained from Table 1 and Table 2. The deviation of δV_oc_ between experiment and theory may not have been due to a low R_sh,_ which can be determined by applying Equation (6), [54], because Table 2 does not support such a hypothesis.

(6) $$\frac{V_{\mathrm{oc}}}{R_{\mathrm{sh}}}=I_{\mathrm{sc}}-I_{o}e^{\left(\frac{{\mathrm{qV}}_{\mathrm{oc}}}{{\mathrm{mkT}}_{c}}\right)}$$

Since the I–V characteristic of M55 module no1 was converted to STC, T_c_ was by principle 25 °C and therefore the additional terms in Equation (5) were zeroed. Introducing the above values into Equation (5) and substituting δV_oc_ = 0.88–0.525 yielded δΙ_ο_/Ι_ο_ = 0.115 or an 11.5% increase due to extended EVA/cell-interface delamination, which is evident in the EL images of module no1 (Section 3.2.1). Therefore, Equation (5) predicts the total δV_oc_ due to any possible set of deviations of the electrical parameters very close to the experimentally determined value.

The aforementioned gradual drop of δI_sc_ = 0.15 A between 5–10 V (Figure 9a) corresponded to R_sh_ and to a shunt diode due to current mismatch attributed to the EVA/cell-interface delamination spots and to the two cells exhibiting different degrees of browning (Figure 9c). This current mismatch caused a low T_c_ pattern when operating in field conditions, which slightly reduced V_oc_ and V_m_. In addition, the cracks, small breaks, and EVA/cell-interface delamination shown in Figure 9b and disclosed by the EL analysis below contributed to δI_m_, as well as an increase of δR_s_ = 0.85 (from Table 2) − 0.30 (manufacturer) = 0.55 Ω and a decrease in R_sh_ to be identified by EL imaging and analysis of the deviations of electrical parameters. Both δR_s_ and δI_m_ = −0.70 A caused a deviation δV_m_ from the nominal value V_m_. 

The expression of the deviation δV_m_ is given by Equation (7), which is another fundamental expression for the study of the deviation of electrical parameters.

(7) $${\delta V}_{m}={\delta V}_{\mathrm{oc}}-\left({\delta I}_{m}R_{s}+I_{m}{\delta R}_{s}\right)$$

Equation (7), using the values from Table 2 and the nominal ones, yielded δV_m_= 1.18 V, and the measured δV_m_ = 17.4 V − 16.15 = 1.25 V, which is a good prediction that also accounts for the degradation in I_m_ and R_s_. The above deviation analysis of the electrical parameters provides a first view of the impact of the defects.

Figure 9d shows the I–V of module no2 with less EVA browning but extended EVA/cell-interface delamination along the busbar. The latter caused an increase in R_s_ and I_o_ and a decrease in R_sh_, confirmed from both Table 2 and the EL image analysis in Section 3.2.1. The values in Table 2, with reference to those in Table 1, yielded δV_oc_ = 0.7 V, δV_m_ = 2.72 V, δR_s_ = 1.43 (Table 2) − 0.30 (manufacturer) = 1.13 Ω, and δI_m_ = 0.366 A. Similar to the above, Equation (3) predicted δV_oc_ = 0.14 V, far less than the 0.7 V determined experimentally. This deviation, according to Equations (5) and (6), may have been due to an increase in the I_o_, that is, a shunt-recombination diode, and to a much lower R_sh_, as it was supported by the higher I_m_ and lower V_m_ compared to module no1. Table 2 confirms this hypothesis. Substituting the above values into Equation (7) yielded δV_m_ = 2.64 V compared to the aforementioned 2.72 V, which demonstrates the very good description of the defects by the above analysis. Modules no1,2 experienced severe external degradation such as deep shading for long periods, which triggered internal defects whose patterns differed, as confirmed by the EL image analysis in Section 3.2.1. Their degradation, as shown in Table 2, was significant, between 25–28%, which is much higher than the 19.2% expected (considering an average degradation of 0.8%/year) for PV modules experiencing only natural ageing and operating for the same number of years. 

**Figure 10:**
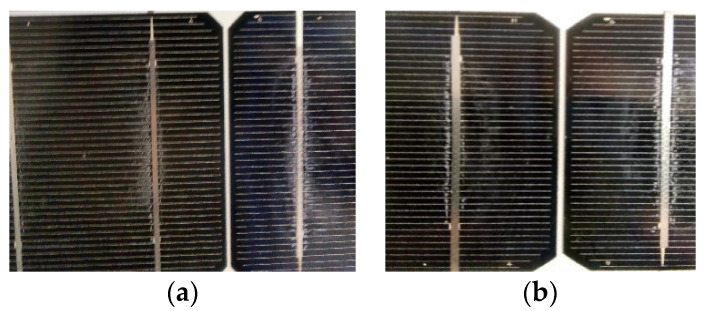
(**a**,**b**) Digital image of SM55 module no3 with extended EVA delamination in the busbar areas.

SM55 no3 showed no EVA browning, and its I–V characteristic is presented in Figure 9e. The module was subject to natural ageing. The EVA/cell-interface delamination was present in all cells, as shown in Figure 10a,b, but it was not as extended as in module no2. Indeed, in Table 2, module no3 showed the least increase in R_s_ compared to no1 and no2 and the smallest P_m_ degradation of 16.8%. The deviation of its electrical parameters was estimated as δV_oc_ = 0.62 V, δV_m_ = 1.41 V, δI_sc_ = 0.347 A, δI_m_ = 0.30 A, and δR_s_ = 0.134 Ω. Equation (7) predicted δV_m_ = 0.94 V, which is much lower than the experimentally determined one, although recombination and delamination effects were taken into account. In this case, the cause of the deviation was the numerous cracks and the grid-line interruptions revealed through EL imaging (Section 3.2.1), whose effect may not be included in Equation (7). This remark allows for the prediction of breaks and cracks in the cells when the δV_m_ estimated by Equation (7) deviates significantly from the measured δV_m_ provided that R_sh_ is not significantly degraded.

**Figure 11:**
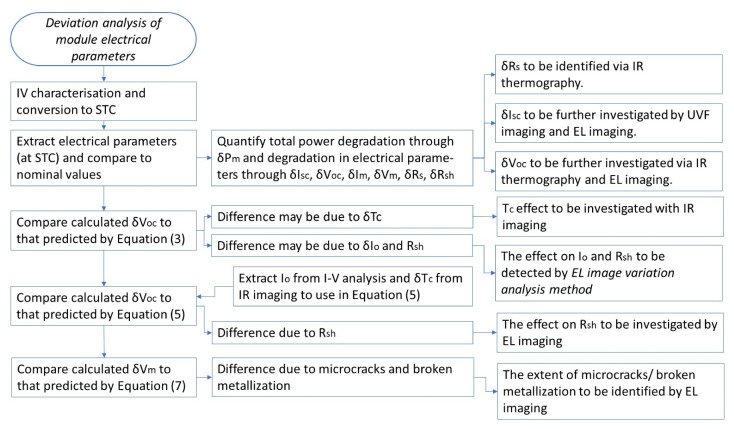
Functional flow-block diagram illustrating the deviation analysis of module-electrical-parameter methodology and synergy with other NDT techniques.

The methodology proposed in the deviation analysis of module electrical parameters is summarized in Figure 11. The synergy with other NDT techniques is also illustrated. Extraction of the electrical parameters from the I–V characteristic can be carried out with any approach, as provided in the review article [55].

### 3.2. The Proposed Diagnostics Component Based on the Variation Analysis of EL Images: Results and Analysis

The second component of the PV-defect diagnostics was based on the analysis of variations of EL images captured in a sequence of bias voltages while measuring the current allowed into the module. The methodology was further supported by IR thermography and UVF imaging and the analysis of deviations of the electrical parameters described in Section 3.1. 

#### 3.2.1. Case 1: c-Si Modules Operating for 18 and 24 Years

**Figure 12:**
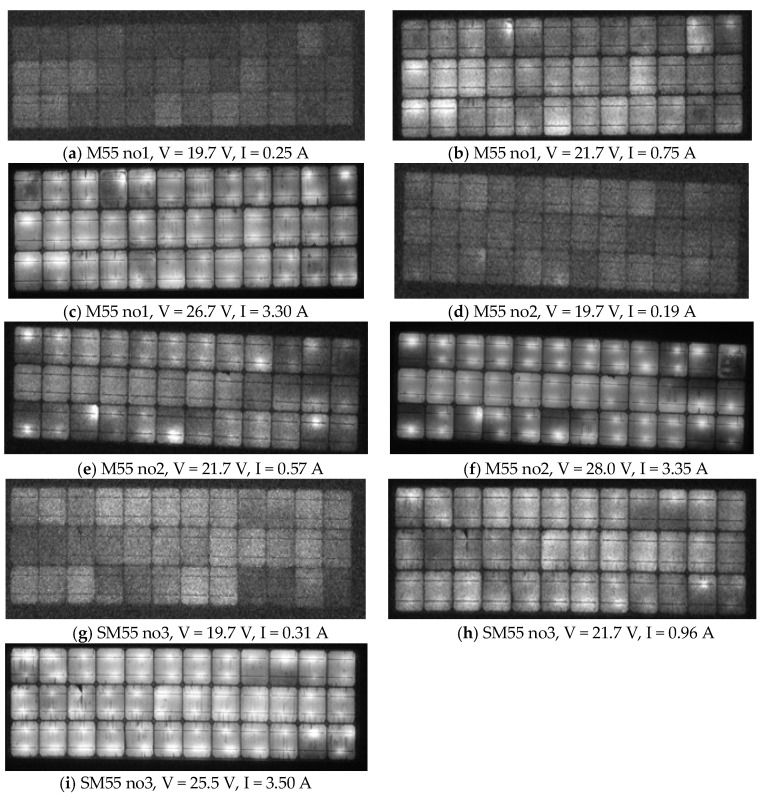
EL images of M55 (**a**–**c**) module no1, (**d**–**f**) module no2, and (**g**–**i**) module no3. The bias V and the allowed I are shown for each case.

The variation in the EL images captured at an increasing sequence of bias V voltages was studied in terms of the strength and pattern of the light/dark contrasts. This could provide significant information about the quality and condition of the module and the type of defects. A number of findings are deployed below that can be considered for a reliable diagnosis of PV defects:

1. Breaks, cracks, and grid-line interruptions in cells were easily identified in Figure 12a–i, which show EL images of the c-Si modules no1,2,3 at various biases V. These defects contributed to I decrease, I–V distortion, R_s_ increase, and R_sh_ decrease. These may have caused small current mismatch. The quantitative effect requires I–V analysis. Cracks in the cell edges may not be identifiable in the UVF images due to photo-bleaching.
2. The dark contrasts in the sequence of EL images captured from low bias V to V = V_oc_ faded away with the increase in the bias V, and this implies that those cells or areas of cells had a lower R_sh_. This is shown in Figure 12a,b,d,e and Figure 12g,h. Such dark contrasts do not appear in healthy cells.
3. The EL images captured for V at around V_oc_ and a little higher showed bright EL spots or areas along the busbar that were attributed to e^−^+hole recombination prevailing at these voltages. This defect increased R_s_, decreased R_sh_, and added a shunt diode. Such cases were numerous in the EL images, as shown in Figure 12, especially along the busbar where EVA/cell-interface delamination was the main defect finding. This did not appear in the EL images of the ODT modules in Section 3.2.2, where EVA/cell-interface delamination was not observed. However, bright spots in the EL images may also appear as a result of corrosion in the busbar, as in Figure 12b (cell position 4 from the left in the upper row), which corresponds to the cell with EVA browning and busbar corrosion in Figure 2c. In module no2, bright EL spots started appearing at lower V *(*Figure 12d), interpreted as damage of the p-n junction with a consequent decrease in V_m_.
4. EL images captured at bias V > V_oc_ may show that dark cell contrasts reduced or disappeared, which demonstrates the presence of cell regions with δR_s_. If the dark contrast is sustained for any V >> V_oc_ it implies cracks, breaks, holes, grid-line interruptions, or inactive regions in cells. Those defected cells contributed to an increase in R_s_ and decrease in R_sh_ and I_sc_ and are numerous in Figure 12, but limited in the ODT modules (Section 3.2.2) corresponding to healthier modules. The overall R_s_ and R_sh_ of the module was determined quantitatively by the I–V analysis, whereas the increase in the R_s_, δR_s_ can be estimated as described in point 7 below.
5. In Figure 12c,f,i with V > *V_oc_* some dark regions in cells were sustained. This implies regions with δR_s_. Figure 12g–i show a more uniform EL illumination pattern. The dark regions in Figure 12g were due to degraded R_sh_ and were more numerous than in Figure 12a. Hence, R_sh_ of no3 was lower than that of no1. At bias V > V_oc_ in Figure 12i, the dark regions were due to δR_s_. In no3 there were fewer than in no1 and much fewer than in no2. That is, the R_s_ in no3 was lower than in no1 and no2. The above statements are in agreement with Table 2.
6. The EL intensity was spatially more uniform in no3 compared to no1 and no2 at bias V > V_oc_ in Figure 12c,f,g. In that V range, the R_s_ governed current I through the cells. Specifically, I vs. V was higher in no3 than no1 and no2, which implies that R_s_ was lower in no3 and higher in no2, which is in agreement to Table 2. The reverse is also true. In high-bias V the effectiveness in defect identification became poorer because the EL luminosity contrast reduced in almost all cells (Figure 12c,f,i).
7. A combination of the deviations δV_oc_, δV_m_, and δR_s_ with the variations of the EL images captured at bias V where the allowed current I = I_sc_ have enriched the proposed defects diagnostics and shed light on the type of defects in the module. In general,
  δV_oc_ = V_bias_ − V_oc,nom_ ≥ I_sc_·δR_s_ (8)
  δR_s_ = R_s,STC_ − R_s,nom_ (9)

The equality in Equation (8) holds when the defects contribute only to increases in R_s,_ e.g., corrosion. The inequality holds when the modules apart from the δR_s_ suffer from increased localised e^−^+hole recombination, EVA/interface delamination, and/or R_sh_ to be confirmed by the sequence of EL image-variation analysis (Figure 12a–c). In addition, the deviation analysis of V_m_ yields:

V_m,nom_ − V_m,STC_ = I·δR_s_ (10)

where I is the current allowed in the module. M55 module no 1 data—V_oc,nom_ = 21.7 V, V_bias_ = 26.7 V, allowed I = I_sc_ = 3.3 A (Figure 12c), R_s_ = 0.85 Ω (Table 2), and R_s,nom_ = 0.30 Ω (manufacturer)—were introduced in Equations (8) and (9). Equation (8) predicted δR_s_ = 1.52 Ω, whereas Equation (9) yielded δR_s_ = 0.85 − 0.30 = 0.55 Ω. This large difference is due to the existence of other defects such as R_sh_ and widely increased e^−^+hole recombination regions (Figure 12c). In addition, introducing module no1 data—V_m,nom_ = 17.4 V and V_m,STC_ = 16.15 V—into Equation (10) yielded δR_s_ = 0.50 Ω, which is very close to the calculation from Equation (9). Similar analysis may be applied to module no2, where Equation (8) predicted δR_s_ = 1.91 Ω, whereas Equation (9) yielded δR_s_ = 1.13 Ω. This large difference is due to the low R_sh_ confirmed by Table 2 and due to increased e^−^+hole recombination. In this case, Equation (10) predicted δR_s_ = 1.01 Ω, which is very close to the value provided by Equation (9), equal to 1.13 Ω.

#### 3.2.2. Case 2: pc-Si Modules Operating for 2 to 5 Years

**Figure 13:**
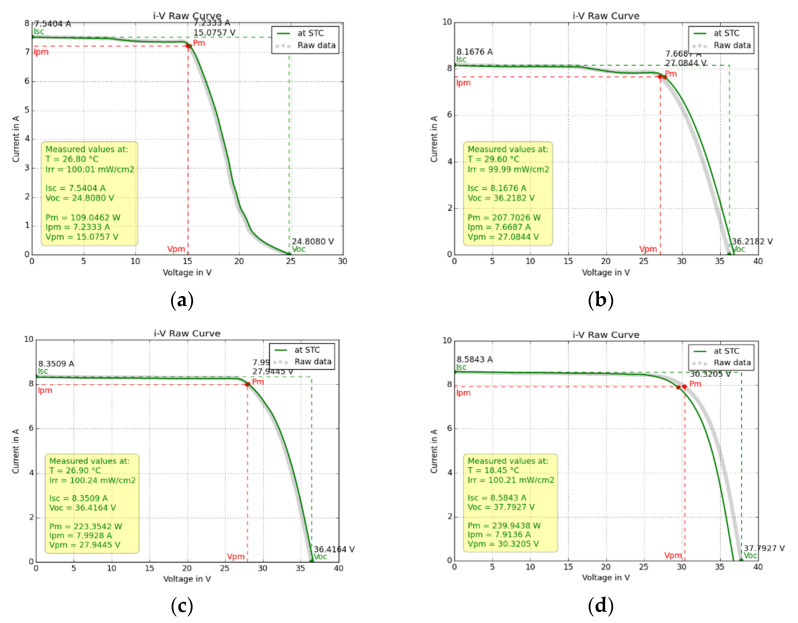
(**a**) The I–V of pc-Si ODT no1, extremely deformed due to organic-film deposition; (**b**) the I–V of the same module recovered from degradation; (**c**) the I–V of ODT module no2 of the same type as no1, (**d**) the I–V of the same type of module no3.

**Figure 14:**
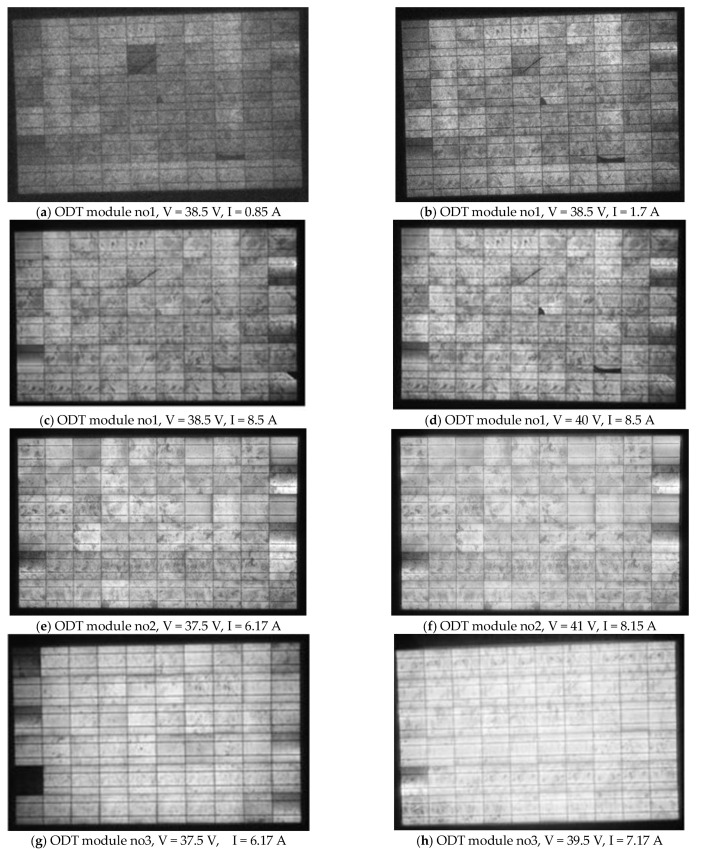
EL images of 3 pc-Si ODT modules that operated for (**a**–**d**) 5 years, (**e**,**f**), 4 years, and (**g**,**h**) 2 years and experienced different degradation effects. The bias V and the allowed I are shown for each case.

Figure 13a–d and Table 3 show the I–V analysis results and Figure 14a–f the EL images of three pc-Si ODT modules with nominal values given in Table 1. Figure 13a shows the I–V of the ODT pc-Si no1 operating for 5 years. Its power degradation was determined to be 52% and the results are given in Table 3. The 12 V decrease in V_oc_ signalled that a string of cells was off. The root cause was a semi-transparent thin film 1.0 g/m^2^ of volatile organic effluents naturally and unevenly deposited on the PV glass. That caused strong current mismatch and crucial I–V deformation. The high R_s_ and low R_sh_ values in Table 3 are the macroscopic results of the I–V analysis and do not reflect the internal conductivity paths in the cells. With the glass cover cleaned, the I–V (Figure 13b) showed power recovery, where the degradation was now 5.9%, still higher than the average anticipated degradation of 4% for a naturally aged module operating for the same number of years (5). The R_s_ and R_sh_ of the cleaned module were consistent with the normal values. ODT no2 and no3 in operation for 4 and 2 years, respectively, showed better I–V profiles, which translated to STC (Figure 13c,d) yielding 2.4% and 1% degradation, respectively.

The EL images of ODT no1 (Figure 14a–d) revealed multiple broken cells, at position (x,y): (6,3), (8,5), (10,6), which remained dark at all bias V. In cell (5,2), a tick sign remained at all bias V, which demonstrates a crack. In addition, periphery cells whose dark parts remained even at higher bias-*V* values were revealed. This implies a δR_s_, and therefore the R_s_ was expected to be higher and the R_sh_ lower than in module ODT no2, as confirmed in Table 3, with no broken parts, as shown in the EL image (Figure 14f). The contrast in the dark parts in some periphery cells and in cell (5,2) decreased when the bias V increased. This implies that those dark regions were due to R_sh_. Figure 14e–h correspond to ODT no2 and no3 and exhibited similar effects of dark regions, which reflects issues related to the manufacturing process. ODT no3 had lower R_s_, as there was only one dark area in the EL image (Figure 14g,h) that remained relatively dark at high bias *V*. This is confirmed in Table 3. Indeed, R_sh_ of no3 was lower than of no1 and no2, as expected, because the cells whose dark contrast decreased with *V* were more numerous than in the other two modules (Figure 14a–f). 

The state of EVA degradation for the ODT modules (Figure 6a–d) with only a few years of operation compared to that of (S)M55 modules (Figure 2c,d) appeared to be healthier, as no EVA browning or delamination in the EVA/cell interface or grid-line interruptions were demonstrated. ODT no1 experienced higher EVA degradation compared to ODT no2 and no3 due to back-sheet delamination caused by stress loads on its large surface. When cleaned from the film of organic-gas effluents its P_m_ recovered greatly. The R_s_ of ODT module no3 was determined to be 0.41 Ω, whereas for modules no1 and no2 it was 0.54 Ω and 0.465 Ω, respectively, which corresponds to about 7–9 mΩ/cell. On the contrary, the R_s_ in the smaller cells of M55 modules was 20–25 mΩ/cell, compared to 18 mΩ/cell for a brand-new module, whereas in modules suffering from several defects and with extended delamination, e.g., M55 module no2, the R_s_ was determined to be equal to 40 mΩ/cell. This shows the effect of natural and induced degradation of the cell’s electric parameters and power performance. 

**Figure 15:**
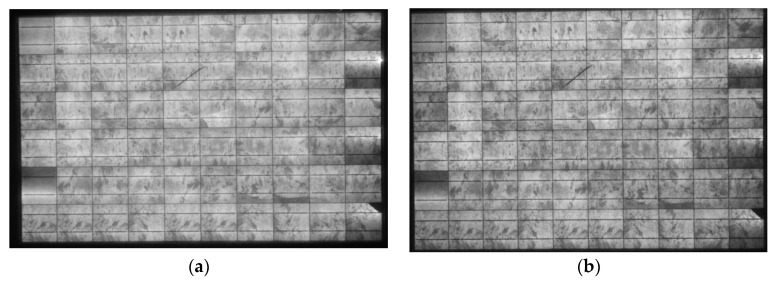
(**a**,**b**). ODT pc-Si module no1 EL images at bias (**a**) V = 42.5 and (**b**) V = 39.5 V. In (**b**), a bright EL spot appears at the edge of the cell in the right column due to a problem in the passivation, whereas in (**a**), the passivation problem is evident in the same cell.

Passivation issues were also disclosed through EL imaging at high bias voltages. the EL image of the ODT pc-Si module no1 at bias V = 42.5 V (Figure 15a) shows that one of the cells in the rightmost column emitted light due to a break in the passivation when the V > V_oc_ by more than 2 V. This defect started at V = 39.5 V (Figure 15b). 

**Figure 16:**
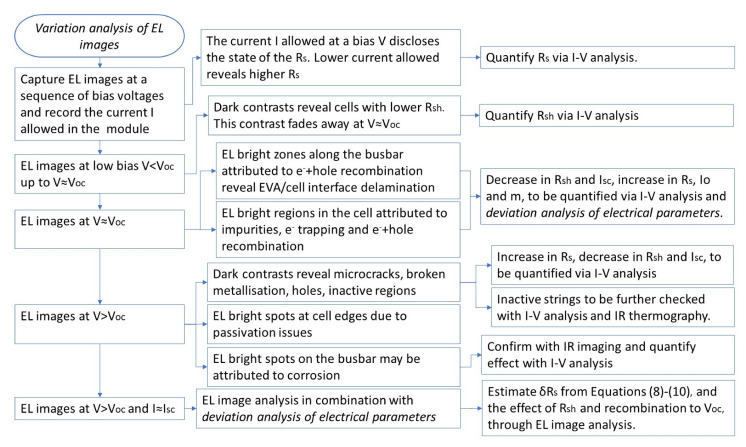
Functional flow-block diagram illustrating the variation analysis of EL images at various bias-voltages and the synergies with other NDT techniques.

The proposed methodology on the variation analysis of EL images at various bias voltages is summarised in Figure 16. The synergy with other NDT techniques is also illustrated.

#### 3.2.3. Discussion

Cross-correlation between the findings from the NDT tool revealed the prevailing defects, their strength, and the effect on PV performance. A diversity of defects and degradation factors was disclosed in the groups of PV modules studied.

The intensity and spatial distribution of the UV fluorescence was directly associated with the degree of EVA degradation, which is proportional to SRD. EVA degradation appeared in UVF images at SRD > 4 MWh/m^2^ for natural ageing, whereas EVA browning appeared at SRD > 10 MWh/m^2^.

PV cells shaded for a long time exhibited high T_c_ profiles due to current mismatch, which caused permanent defects: (a) EVA browning (directly disclosed by UVF and optical inspection and indirectly by I–V in cross-correlation with IR), (b) delamination of EVA/cell interface (disclosed by optical inspection and high-resolution EL imaging), and (c) corrosion on the busbar or in the cell interconnects (disclosed by IR thermography and indirectly by I–V analysis).

The e^−^+hole recombination due to impurities may occur even in brand new modules. T_c_ profiles of 10–20 °C higher than the temperature of neighbouring cells appeared in the IR thermography due to current mismatch. This was also confirmed by studying the EL image and indirectly by I–V-profile analysis. EVA browning can spatially affect the light transmission into the cell and cause current mismatch. This implies a shunt diode, a δR_sh_, and high T_c_ profiles, which trigger second-order generation defects such as wider and/or deeper EVA browning, and may also lead to delamination and corrosion.

There is a time evolution in the defects analysed, as they depend on SRD and any casual external factors, causing high T_c_ profiles and second-order generation defects, which form avalanches. These defects may not be predictable or predetermined. More crucial than the P_m_ degradation is the I_m_ and V_m_ decrease, which causes mismatch between the strings of modules.

Film deposition on the module, breaks, cracks, grid-line interruptions, R_s_ and R_sh_, e^−^+hole recombination centres, and cell-edge passivation issues were demonstrated by studying the variation of EL images captured under a series of bias voltages and the deviation analysis of the electrical parameters of the module using I–V data analysis. In addition, UVF and IR supported the identification of those defects. Prediction of the diversity of families of defects was possible based on the diagnostics methodology developed for the defects.

## 4. Conclusions

This paper provides an extended analysis on PV-defect identification in cells and modules based on the synergy of NDT tools, UVF imaging, IR thermography, EL imaging, and I–V analysis. A large number of PV modules operating for different years, from 0 to 24 years, was studied, as they exhibit a wide range of defects. The defects diagnosis is based on the cross-correlation of the findings from the above NDT techniques. It determines the defects qualitatively and quantitatively as concerns their spatial pattern, the strength of their effect, and the performance degradation. A methodology based on the synergy of NDT techniques is proposed with the following two main components focusing on the quantitative and qualitative analysis of defects:

1. Analysis of the deviations of the module electrical parameters at STC from their nominal values of δV_oc_, δV_m_, δI_sc_, δI_m_, δR_s_, and δR_sh_. This provides an insight into the impact of defects on the module electrical parameters, along with an inference on the origin of the defect to be further identified in synergy with the aforementioned NDT tools and the second component of this methodology.
2. Variation analysis of EL images captured in a sequence of bias voltages, from V < V_oc_ to V > V_oc_, and measuring the current allowed into the module. The analysis discloses regions in the cells with δR_s_, δR_sh_, shunt diodes, passivation issues, e^−^+hole recombination centres, holes, cracks, breaks, grid-line interruptions, and further defects identified in synergy with other NDT techniques. The quantification of the impact of defects is further supported by I–V analysis and the first component of this methodology.

Additionally, this research contributes further to defect diagnosis through the model proposed for the temperature effect of the current mismatch between cells in a module and hotspot formation due to corrosion, supported by IR thermography. Furthermore, mathematical models for the quantification of the deviations ${\delta V}_{\mathrm{oc}}$ and ${\delta V}_{m}$ are proposed within the methodology developed for the deviation analysis of the module electrical parameters.

The methodology demonstrated that the external and internal degradation factors and defects, depending on their strength and the environmental and operating conditions, create second-order defects, expanding in space and time and advancing on a cause-and-effect process like an avalanche. Subsequently, the degradation rate is not a linear function of time and increases more quickly when families of defects are developed. This PV-diagnostics methodology using synergistic NDT tools provides reliable detection of defects and quantitative determination of their impact on the module electrical parameters and would be very useful for the development of a future online monitoring and diagnostics system.

## Figures and Tables

**Table 1:** Nominal values of c-Si and pc-Si modules used in the diagnostics methodology.

Module	I_sc_ (A)	V_oc_ (V)	I_m_ (A)	V_m_ (V)	P_m_ (W_p_)
M55 c-Si no1 and 2	3.35	21.7	3.05	17.4	53.0
SM55 c-Si no3	3.45	21.7	3.15	17.4	54.8
ODT pc-Si no1 and 2	8.45	36.9	7.84	29.4	230
ODT pc-Si no3	8.56	37.1	7.95	29.6	235

**Table 2:** The electrical parameters, converted to STC, of two c-Si modules operating for 24 years with induced ageing from extended shading and another c-Si module operating for 18 years under natural ageing.

Modules c-Si	I_sc_ (A)	V_oc_ (V)	I_m_ (A)	V_m_ (V)	R_s_ (Ω)	R_sh_ (Ω)	P_m_ (W)	δP_m_/P_m_ %
M55 no1 24 years	2.550	20.82	2.496	16.15	0.85	106.7	38.71	28.0
M55 no2 24 years	2.964	21.0	2.684	14.68	1.43	71.4	39.55	25.4
SM55 no3 18 years	3.003	21.08	2.850	15.99	0.794	103.9	45.57	16.8

**Table 3:** The electrical parameters, converted to STC, of 3 pc-Si ODT modules. The results for ODT module no1 are given twice, for the uncleaned module and with its glass cover cleaned.

Modules pc-Si	I_sc_ (A)	V_oc_ (V)	I_m_ (A)	V_m_ (V)	R_s_ (Ω)	R_sh_ (Ω)	P_m_ (W)	δP_m_/P_m_ %
ODT no1 5 years	7.53	24.59	7.22	15.26	3.0	60	110.2	52
ODT no1 cleaned	8.48	36.50	8.00	27.00	0.54	151	216.4	5.9
ODT no2 4 years	8.317	36.67	8.012	28.04	0.465	149	224.6	2.4
ODT no3 2 years	8.61	36.80	7.89	29.50	0.410	145	232.7	1.0

## Data Availability

The data presented in this study are available on request from the corresponding author.

## Nomenclature

A_c_	PV-cell area (m^2^)	T_f_: T_b_	PV-module temperature in the front and back side, respectively (K)
A_cor_	I area in a cell with corrosion (m^2^)	T_pv_, T_c_	PV-module and -cell temperature, respectively (K). Ideally equal.
ARC	Anti-reflective coating	U_f_, U_b_, U_pv_	The heat-loss coefficient in the front side, back side, and the whole module, respectively (W/m^2^K)
EL	Electroluminescence	UVF	Ultraviolet fluorescence
EVA	Ethylene–vinyl acetate	V_d_	Voltage across the bypass diode in the module
I, I_m_, I_sc_	The current in a PV module, the current at maximum power point, and the current at short circuit, respectively (A)	V_m_, V_oc_	PV-module voltage at maximum power point and at open circuit, respectively (V)
I_o_	Reverse saturation current (A)	V_oc,c_	PV-cell voltage at open circuit (V)
I_ph_	Photocurrent (A)	h_c_, h_r_	Coefficients due to heat convection and IR radiation at the front side of the PV module (W/m^2^K), represented by h_c,f_ + h_r,f_
I_T_	In-plane solar irradiance (W/m^2^)	h_c,f_,h_c,b_	Heat-convection coefficient of the front and PV back surface to air (W/m^2^K)
IR	Infrared	h_r,f_,h_r,b_	Radiative-heat coefficient of the front and back side of the PV to environment (W/m^2^K)
NDT	Non-destructive testing	k	Boltzmann constant 1.3806488 × 10^−23^ J/K
P_m_	PV output at maximum power point (W)	m	Ideality factor of the PV-cell diode
PL	Photoluminescence	n_s_	Number of cells in series in the module
R_s_	PV-module series resistance (Ohm)	q	Electron charge 1.602 × 10^−19^ C
R_sh_	PV-module shunt resistance (Ohm)	v_w_	Wind speed on the PV module (m/s)
SRD	Solar radiation dose (MWh/m^2^)	δI_m_, δI_sc_, δΙ_ο_	The deviation of I_m,_ I_sc_, and I_o_ at STC from their nominal values, respectively (A)
STC	Standard test conditions (I_T_ = 1000 W/m^2^, AM1.5,T_c_ = 25 °C)	δV_m_, δV_oc_	The deviation of V_m_ and V_oc_ at STC from their nominal values, respectively (V)
SWIR	Short-wave infrared	δR_s_, δR_sh_	The deviation of R_s_ and R_sh_ at STC from their nominal values, respectively (Ohm)
T_a_	Ambient temperature (K)	η	PV efficiency
